# Wettability of Magnetite Nanoparticles Guides Growth from Stabilized Amorphous Ferrihydrite

**DOI:** 10.1021/jacs.1c02687

**Published:** 2021-07-15

**Authors:** Lucas Kuhrts, Sylvain Prévost, Daniel M. Chevrier, Péter Pekker, Oliver Spaeker, Mathias Egglseder, Jens Baumgartner, Mihály Pósfai, Damien Faivre

**Affiliations:** † Max Planck Institute of Colloids and Interfaces, Am Mühlenberg 1, 14476 Potsdam, Germany; ‡ Institut Laue-Langevin, 71 avenue des Martyrs, CS 20156, 38042 Cedex 9 Grenoble, France; § CNRS, CEA, BIAM, Aix-Marseille University, 13108 Saint-Paul-lez-Durance, France; ∥ Research Institute of Biomolecular and Chemical Engineering, University of Pannonia, Egyetem u. 10, H8200 Veszprém, Hungary

## Abstract

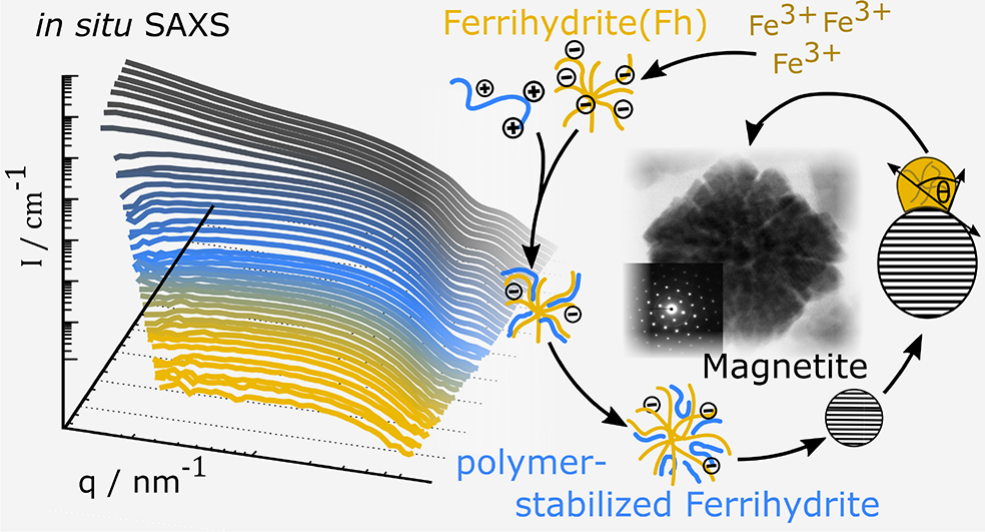

Crystal formation via amorphous precursors is a long-sought-after gateway to engineer nanoparticles with well-controlled size and morphology. Biomineralizing organisms, like magnetotactic bacteria, follow such a nonclassical crystallization pathway to produce magnetite nanoparticles with sophistication unmatched by synthetic efforts at ambient conditions. Here, using *in situ* small-angle X-ray scattering, we demonstrate how the addition of poly(arginine) in the synthetic formation of magnetite nanoparticles induces a biomineralization-reminiscent pathway. The addition of poly(arginine) stabilizes an amorphous ferrihydrite precursor, shifting the magnetite formation pathway from thermodynamic to kinetic control. Altering the energetic landscape of magnetite formation by catalyzing the pH-dependent precursor attachment, we tune magnetite nanoparticle size continuously, exceeding sizes observed in magnetotactic bacteria. This mechanistic shift we uncover here further allows for crystal morphology control by adjusting the pH-dependent interfacial interaction between liquidlike ferrihydrite and nascent magnetite nanoparticles, establishing a new strategy to control nanoparticle morphology. Synthesizing compact single crystals at wetting conditions and unique semicontinuous single-crystalline nanoparticles at dewetting conditions in combination with an improved control over magnetite crystallite size, we demonstrate the versatility of bio-inspired, kinetically controlled nanoparticle formation pathways.

## Introduction

The theory of nonclassical nucleation conceptualizes the formation of solids via condensed, noncrystalline transient particles. It has been demonstrated in numerous examples that such prenucleation cluster-driven processes can produce a variety of complex synthetic crystal architectures.^1^ Similar strategies are employed in biomineralizing organisms, where hybrid materials with tailored properties^2,3^ are realized by a spatially controlled mineralization process directed through additive-stabilized amorphous precursors.^4−7^ Such biomineralization pathways are the benchmark for the preparation of synthetic systems with equally refined properties at ambient conditions.^8,9^ Magnetotactic bacteria, for example, produce magnetite (Fe_3_O_4_) nanoparticles precisely defined in size and morphology.^10^ Intrigued by the biomimetic effect of poly(arginine) additives on magnetite nanoparticle formation, we envision that the presence of this simple polypeptide may impose a biomineralization analogous pathway to obtain equal control over the crystal properties.^11^ Although biomineralized magnetite crystals are formed under environmental conditions via a transient, protein-stabilized ferric (oxyhydr)oxide^12,13^ and similar amorphous precursors have been observed in synthetic magnetite,^14^ no approach to rationalize and direct their crystallization has yet emerged.

The enhanced control in nonclassical crystal formation is founded on the formation of kinetically favored amorphous precursors and their cascade-like solid-state transformation to the thermodynamically most stable crystalline phase. Control over crystal growth can be induced by altering the energy landscape of each involved solid-state transformation by the presence of macromolecular^15^ or ionic additives.^6^ However, a holistic theory to predict the exact formation pathway has not yet emerged. This may be due to numerous interdependent parameters prevalent in such precursor-driven processes such as precursor attachment reaction kinetics, hydration forces, and interaction energies at the amorphous/organic/mineralized interface.^16^ A better understanding of these contributions will enable us to engineer organic/inorganic hybrid systems as well as purely crystalline nanoscopic systems. However, because of low concentration,^17^ low density, and strong hydration of the involved amorphous precursors, temporally and structurally resolving their involvement in crystallization pathways in their native environment to infer a mechanism remains challenging.

Herein, we study the biomimetic synthesis of magnetite in the presence of the simple polypeptide, poly(arginine), with high brilliance synchrotron small-angle X-ray scattering (SAXS) to observe the growth process *in situ*. In brief, magnetite was coprecipitated in aqueous alkaline solution from ferrous and ferric chloride (molar ratio 1:2) in the presence of poly(arginine) (see section SI1 of the Supporting Information) and scattering patterns recorded at different time points (see section SI4). While pure magnetite is notoriously difficult to synthesize in a controlled manner under ambient aqueous conditions due to the low-concentration supersaturation limits of ferrous and ferric chloride, our results indicate that an uncommon but well-defined nanocrystal morphology forms by the surface-wetting properties of a polymer-stabilized precursor phase in the presence of poly(arginine). This wettability can be quantified with respect to pH, leading to a semicontinuous single crystal at high pH and low wettability.

## Results and Discussion

**Figure 1:**
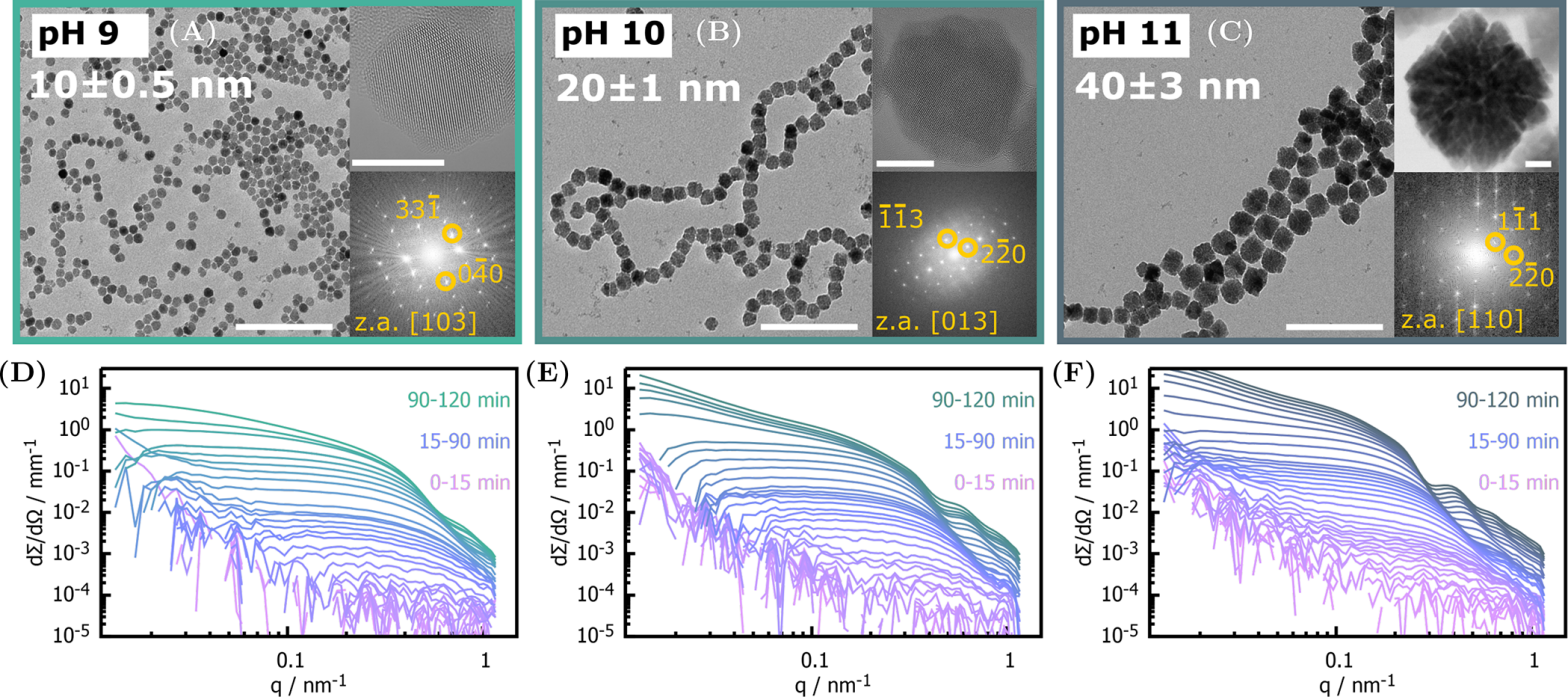
(A–C) Representative TEM micrographs (scale bar 200 nm) for particles grown for 2 h at pH 9, 10, and 11, with corresponding mean sizes. The HRTEM (scale bar 10 nm) images (top right) emphasize a morphological transition between pH 10 (B) and 11 (C) from dense to substructured nanoparticles. Corresponding FFTs (inset bottom right) show single crystalline diffraction and can be indexed according to the inverse spinel structure of magnetite. (D, E) Corresponding time-resolved *in situ* SAXS data of the syntheses at pH 9, 10, and 11. Different colors indicate time windows when different scattering contributions are dominant including amorphous, low-dimensionality iron structures (0–15 min), solid nanoparticles (15–90 min), and chainlike aggregates (90–120 min).

Magnetite nanoparticles formed in the presence of poly(arginine) exhibit a morphological transition from compact (pH 9–10) to substructured single crystals (pH 11), visible in TEM micrographs (Figure 1A–C). Moreover, their size increases with increasing pH, opposed to what is observed for additive free magnetite (Figure SI8). The origin of both effects induced by poly(arginine) remained so far elusive. We thus measured *in situ* SAXS to temporally resolve magnetite formation at pH 9, 10, and 11 (Figure 1D–F). Because of a higher X-ray contrast against water, the scattering signal arises predominantly from condensed iron species, while poly(arginine) remains invisible. We hence observe during the first 15 min (pink curves) the signal characteristic of a sub-10 nm low-dimensionality iron fluctuation. After 30 min (violet curves), intensity oscillations emerge indicative of solid nanoparticles. Finally, particles form chainlike aggregates after 90 min.

**Figure 2:**
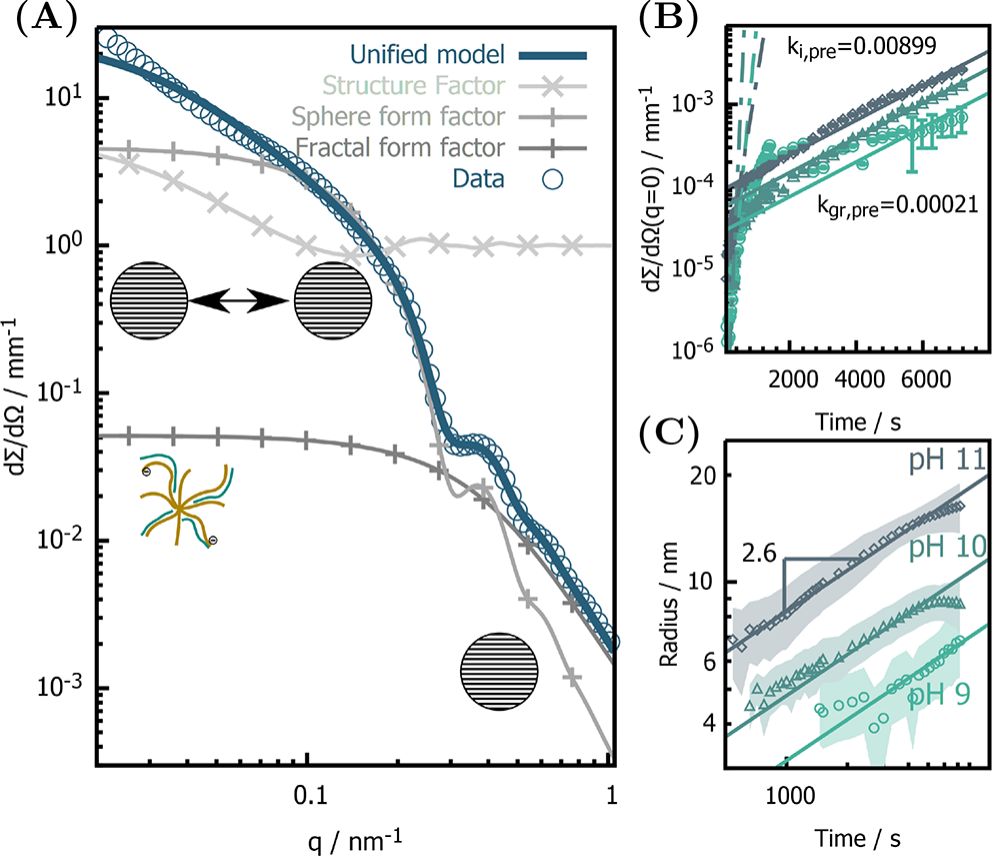
(A) Analytical description of single scattering curve of final product to illustrate contributions of form factors (lines) and structure factor (cross) to unified scattering model (solid line) used to fit all time-dependent SAXS data. Time-dependent parameters, including (B) the forward scattering of precursors, proportional to their concentration, and (C) the radius of solid nanoparticles with shaded areas indicating the standard deviation in particle size. The nanoparticle growth is fitted with a growth law excluding the last data points, where the growth dynamics flattens off. From the fits we obtain the kinetic constant that scales with pH as *k*_R,9_ = 1.25, *k*_R,10_ = 1.40, and *k*_R,11_ = 1.79 nm s^–1/α^ with a consistent growth exponent of 2.6.

**Figure 3:**
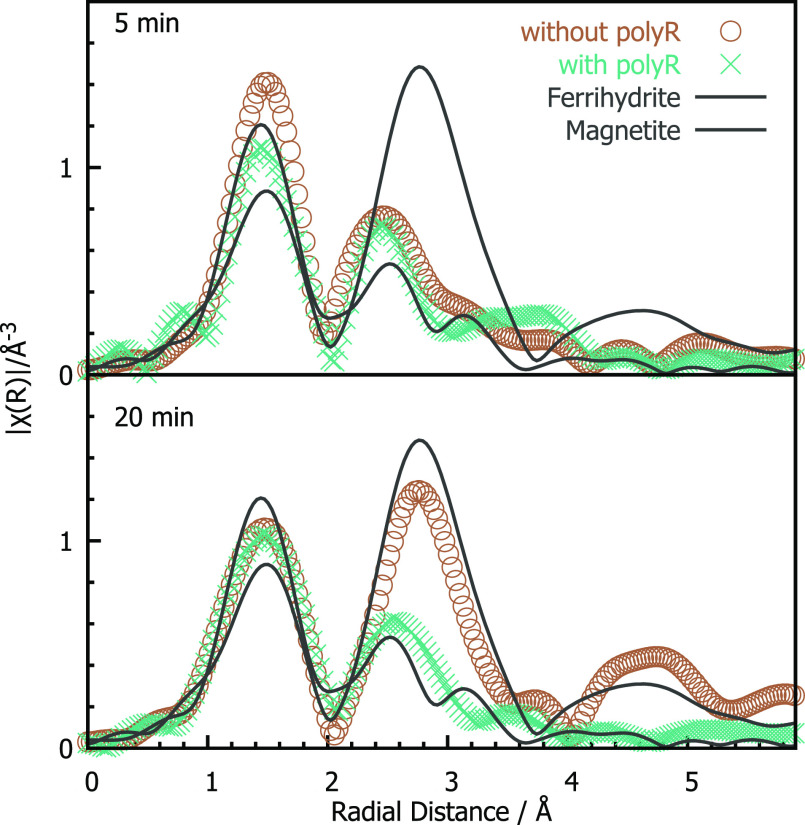
Iron-centered radial distribution functions of aliquots taken from the synthesis after 5 and 20 min with and without the addition of poly(arginine) at pH 11 compared with magnetite and ferrihydrite references. With the addition of poly(arginine), the iron coordination resembles that of ferrihydrite for both time points. In the absence of poly(arginine), ferrihydrite-like iron coordination is observed for the early time point, while after 20 min mostly magnetite is observed.

To retrieve quantitative time-dependent structural information, we used an analytical scattering model (see the Supporting Information for an explanation) to fit the SAXS data. We employ three time-varying scattering contributions to describe the full data set, exemplarily shown for the scattering signal of the last time point of the synthesis in Figure 2A. In brief, all *in situ* SAXS data (Figure SI4) can be fitted with three scattering contributions. First, we modeled a time-varying contribution of a solid spheres form factor, from which we retrieve concentration and size (Figure 2C) of the magnetite nanoparticles. The second contribution originates from amorphous low-dimensionality iron fluctuations that are best described by a fractal form factor^18^ with a time- and pH-independent correlation length of around 4 nm and a fractal dimension of 2.2, showing a time-varying concentration (Figure 2B). At later stages, the third contribution from chainlike aggregates are modeled with a pair potential structure factor. The temporal evolution of the amorphous, low-dimensionality iron fluctuation concentration (Figure 2B) indicates their role as precursors in the formation of magnetite; their concentration indeed increases for all pH conditions with a first-order reaction kinetic constant of *k*_i,pre_ = 0.089 nm/s and is decreased after 300 s to a rate of *k*_gr,pre_ = 0.002 nm/s. This decrease correlates with the appearance of nanoparticles (Figure SI9), demonstrating that the low-dimensionality iron fluctuation is consumed in the formation of nanoparticles qualifying them as a transient precursor phase. Because of the consistent retardation of precursor concentration in combination with constant nanoparticle number density (Figure SI5C), we infer that the nanoparticles grow by a discrete addition of precursors, which is independently confirmed by a model-free SAXS data analysis (Figure SI5A,B).^19^ SAXS data further resolve the nanoparticle size as a function of time (Figure 2C), which can be used to identify the growth mechanism. We fit the growth for all pH with the rate law *R*(*t*) = *k*_R_·*t*^1/α^, yielding the growth rate *k*_R_ and the growth exponent α from which the growth mechanism can be inferred. While magnetite in the absence of additives exhibits reaction limited growth (α = 1),^20^ we find a significant retardation (α = 2.6) of the growth mechanism when poly(arginine) is added for all pHs. This growth exponent, however, does not correspond to any reported particle growth mechanism. Additionally, while rate constants for pure magnetite scale inversely with pH,^20^ we find an inverse behavior when poly(arginine) is added with *k*_R,9_ = 1.25, *k*_R,10_ = 1.40, and *k*_R,11_ = 1.79 nm s^–1/α^. Both the change in the growth exponent and the inversion of the pH-dependent growth rate indicate a fundamental change in the formation mechanism of magnetite when poly(arginine) is added to the synthesis. To further understand the effect of poly(arginine) on magnetite formation, we studied the iron phases at early (5 min) and later (20 min) time points with and without the addition of poly(arginine). Comparing radial distribution functions obtained from cryo extended X-ray absorption fine structure (EXAFS) measurements at the iron K-edge (Figure 3), we show that both 5 min samples exhibit an iron local structure similar to that in ferrihydrite (higher Fe–O contribution at ≈1.5 Å, lower Fe–Fe contribution at ≈2.5 Å, not phase shift corrected), supported by quantitative linear combination fits shown in Figure SI3. With the addition of poly(arginine), this local structuring persists in the 20 min sample, whereas without the addition of poly(arginine) the local iron coordination resembles more strongly that of magnetite.

**Figure 4:**
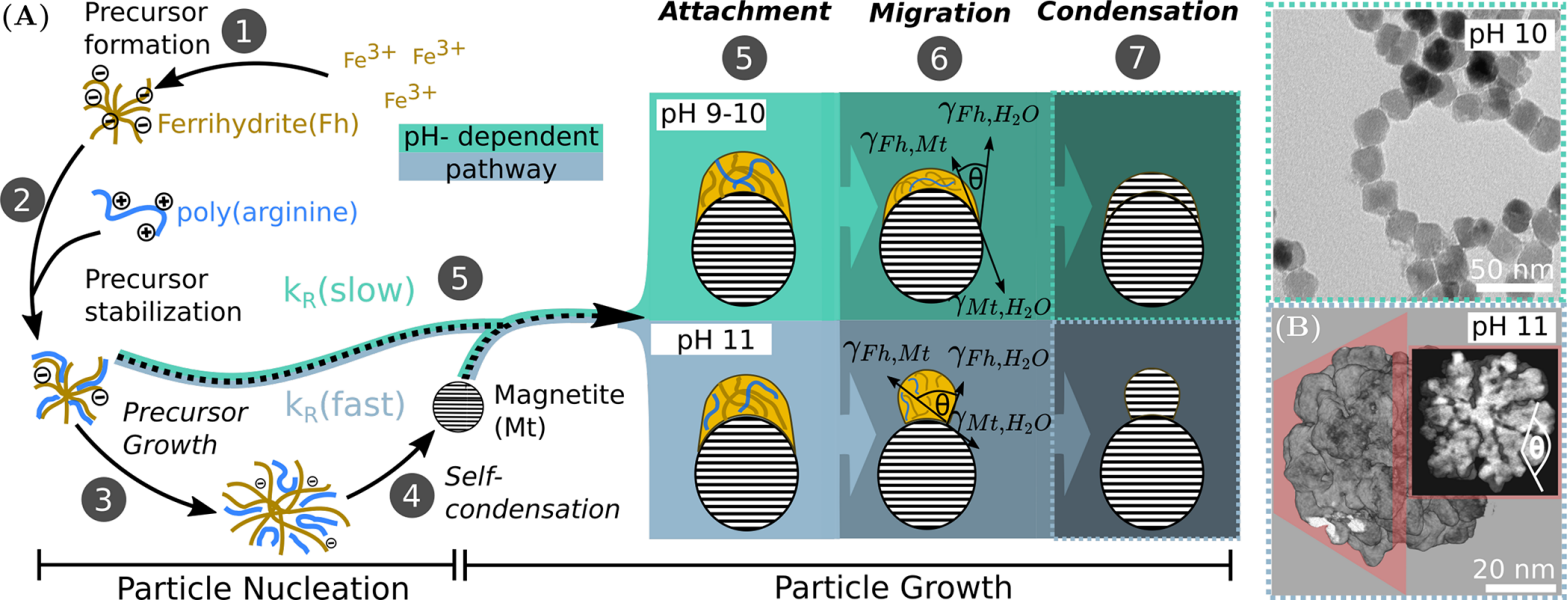
(A) Proposed mechanisms of magnetite (Mt) formation in the presence of poly(arginine), including the precipitation of the ferrihydrite (Fh) precursor (1) and its stabilization by poly(arginine) (2) prior to the growth-induced (3) self-condensation (4) to form a magnetite seed particle. We identify three steps that determine the final particle size and morphology: (5) the pH-dependent precursor attachment, quantified by the rate constant *k*_R_, (6) the pH-dependent precursors migration, determining the final morphology (compact at pH 9–10 or substructured at pH 11), and (7) the precursor condensation, inducing single-crystalline properties via homoepitaxy. The extent of precursor migration, which is dependent on the surface energy of ferrihydrite at the magnetite/water interface, is quantified by using the Young–Laplace equation, γ_Mt,H_2_O_ = γ_Pre,Mt_ + γ_Pre,H_2_O_ cos(θ). At higher alkalinity, the contact angle, θ, increases due to a surface charging of ferrihydrite and magnetite. The wetting-induced structure is fixed during the magnetite/ferrihydrite solid-state transformation. (B) From a 3D reconstruction obtained from HAADF STEM tomography, a particle obtained at pH 11 is virtually sliced (inset) revealing subunits crystallized at high contact angles, highlighting the discontinuous nature of the formed particles.

We propose a formation mechanism (Figure 4) for the biomimetic magnetite nanoparticles originating from a polymer-stabilized, amorphous ferrihydrite-like precursor exhibiting a pH-independent structure (Figure 4A, 1–2). A similar precursor phase has been proposed—but never experimentally detected—for the coprecipitation reaction to form magnetite^20,21^ and under low-pH, low-driving-force conditions for phase separator in iron solutions.^22^ Equally, for the biomineralization in magnetotactic bacteria ferrihydrite-like precursors have been identified.^12,13^ EXAFS data suggest a polymer-induced kinetic stabilization of the ferrihydrite against condensation to magnetite. Such metastability of transient ferrihydrite precursors has been demonstrated for iron oxides with small nanoparticle sizes and large surface area.^23^ We similarly propose that the presented stabilization is induced by poly(arginine) counteracting the agglomeration of amorphous ferrihydrite particles,^24^ which thus retain a size of 4 nm where ferrihydrite can be considered thermodynamically stable with respect to magnetite.^25^ Consequently, the transformation to a magnetite seed particle (Figure 4A, 3–4) must be induced by crossing a critical precursor size, either by aggregation or by growth.

**Figure 5:**
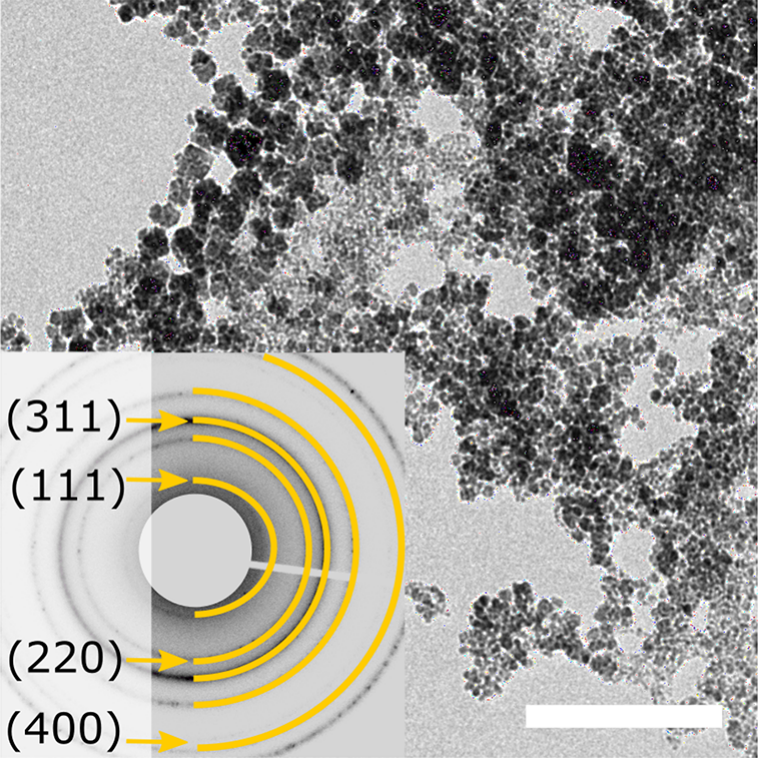
TEM micrograph of magnetite nanoparticles synthesized at pH 11 for 2 h with an initial addition of 10 mM NaCl. Nanoparticle morphology resembles that of additive-free magnetite. The electron diffraction patterns (inset) is indexed according to the inverse spinel structure of magnetite. Scale bar represents 200 nm.

The formed magnetite seeds further grow by precursor attachment (Figure 4A, 5) at increased rates at higher alkalinity. As particle diffusion is not dependent on pH, we identify the reaction of precursor attachment as the rate-limiting step. The increased reaction rate (*k*_R_) at higher alkalinity can thus be deduced from the catalysis of the involved nucleophilic oxolation reaction.^14^ Thermodynamics predicts a slower growth of additive-free magnetite at increased alkalinity due to a lowering of the magnetite/water surface tension decreasing the critical size at which a crystal seed is considered stable^17,20,26,27^ (a more detailed description can be found in section SI3.1) Oppositely, the addition of poly(arginine) induces a kinetically controlled growth that is accelerated at higher pH. The inversion of the pH-dependent growth rates can thus be interpreted as a shift from a thermodynamically controlled reaction in the absence of poly(arginine)^20^ to a kinetically controlled reaction in the presence of poly(arginine).^28^

**Figure 6:**
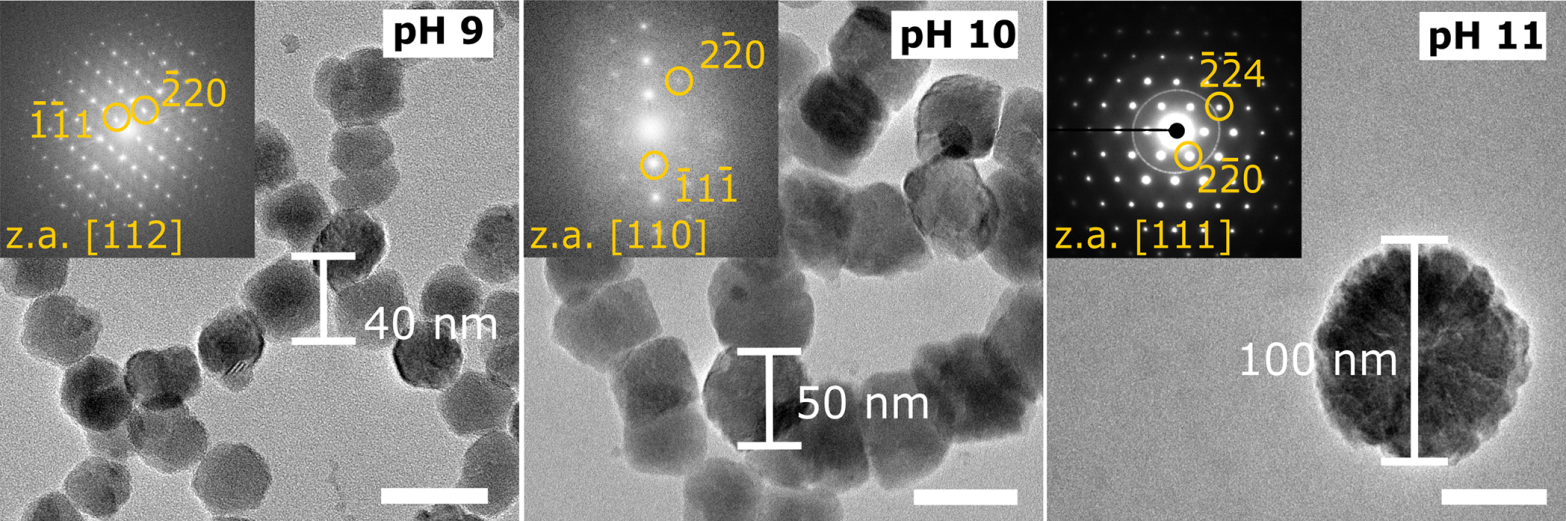
TEM images (scale bar 50 nm) of particles produced by redesigned synthesis protocol, grown for 20 h at pH 9, 10, and 11 (A–C) reaching sizes of 50 nm for solid and sizes up to 100 nm for substructured nanoparticles. The FFTs/SAED (top inset) were indexed according to the inverse spinel structure of magnetite and show single crystalline diffraction for all pH.

While pH not only affects particle growth rates, it further determines the morphology of magnetite nanoparticles, leading to compact particles at low alkalinity and substructured crystals at high alkalinity. We propose this transition to emerge from a pH-dependent change in interfacial energy between the precursor and the magnetite/water interface. Approximating the ferrihydrite precursor as a liquid,^29^ we treat its interaction with magnetite as a wetting process (schematically shown in Figure 4A, 6). Consequently, the precursor wettability is quantified by using the simplified Young’s relation, relating the contact angle between the magnetite nanoparticle (Mt) and the precursor (pre) to their interfacial tension, γ. An increase in pH increases the surface charge density of ferrihydrite and magnetite by shifting the protonation equilibrium of surface hydroxyl groups. Assuming the change in surface charge changes the surface tensions of magnetite/water (γ_Mt,H_2_O_) and precursor/water (γ_pre,H_2_O_) alike,^30^ we establish a direct proportionality (full derivation from Young’s relation in section SI2.2) between pH and contact angle via
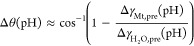
This limited interaction between magnetite and precursor at increased alkalinity—known as dewetting—accordingly allows us to explain all observed nanoparticle morphologies in this study. For pH 9 and 10, a low contact angle induces complete precursor wetting and thus compact nanoparticles. In contrast, the increased γ_Mt,pre_ induces dewetting at pH 11. The shape of the ferrihydrite precursors is preserved during the transformation to magnetite and persists due to the low solubility of magnetite, where atomic rearrangement processes are kinetically hindered. The thermodynamically unfavored substructured morphology is thus conserved in the final product visible in 3D rendered volume reconstruction (Video SI1) obtained from HAADF STEM tomography (Figure 4C). The proposed dewetting leads to bulges of magnetite on the nanoparticle surface (inset, Figure 4C) that are overgrown by consecutively crystallizing precursor, resulting in a semicontinuous crystal exhibiting single-crystal diffraction (FFT inset, Figure 1C). A further increase to pH 12 hampers magnetite/precursor interaction to an extent where precursor self-condensation is favored resulting in predominantly small magnetite nanoparticles (Figure SI8). All observed nanoparticle morphologies exhibit single-crystalline properties originating from the homoepitaxial transformation of the precursor, where ferrihydrite assumes the crystallographic order of its subphase magnetite. Combining the pH-dependent wettability of the ferrihydrite precursors with its homoepitaxial transformation, this mechanism can account for the previously elusive single-crystalline properties of the substructured nanoparticles properties.^31^

Understanding the here-presented formation pathway enables us to lift former synthesis limitations that resulted in a previously reported arrest of nanoparticle growth after 2 h.^31^ Because of the continuous addition of iron chloride to the synthesis vessel, the released counterion (NaCl) concentration increases to 10 mM after 2 h screening the charges of poly(arginine), suppressing its interaction with the precursor.^32^ In a separate synthesis, we demonstrate by TEM (Figure 5) that an initial addition of 10 mM NaCl and other monovalent of counterions (NaBr and KCl, Figure SI6) leads to the predicted formation of nanoparticles with ill-defined morphologies (Figure SI7), similar to what is expected for additive-free magnetite. In a second synthesis approach, we aimed to overcome the presented growth-inhibiting effects of the counterions. This was achieved by increasing the synthesis volume—diluting the released counterions—prolonging for a given iron addition rate the unhindered growth time of the nanoparticles. As a result, we can grow particles for 20 h and obtained compact magnetite nanoparticles at pH 9 and 10 with dimensions up to 50 nm and substructured crystals with dimensions up to 100 nm (Figure 6). Overall, we demonstrate how a bio-inspired approach in combination with a rationally designed nanoparticle synthesis can yield similar control over particle size and morphology as exhibited by magnetotactic bacteria,^33^ establishing the coprecipitation reaction as a synthesis route under ambient, aqueous conditions yielding size and shape control of magnetite comparable to conventional routes requiring elevated temperatures or organic solvents.^34,35^

## Conclusion

In conclusion, we present an *in situ* time-resolved SAXS study monitoring the bio-inspired formation of magnetite nanoparticles from a polymer-stabilized, amorphous ferrihydrite precursor. We thus provide further evidence of the presence of ferrihydrite as a transient precursor in the synthetic coprecipitation of magnetite. The addition of poly(arginine) shifts the reaction control from thermodynamic to kinetic, directing nanoparticle growth via precursor attachment reactions and explaining the elusive inversion of the magnetite size dependence on pH.^17^ The unexpected wetting of the liquidlike precursor and the consecutive homoepitaxial crystallization induce single-crystalline properties. These properties are even observed within the substructured, discontinuous nanoparticles obtained at high pH, which—not complying to the classification of a mesocrystal^36^—can be regarded as semicontinuous single crystals. From this work, we demonstrate how a bio-inspired kinetic pathway can be rationalized to attain control over nanoparticle formation, leading to continuously tunable nanoparticle size with adjustable morphology. Underlining the predictive nature of the presented mechanism, we were able to overcome previously encountered particle size limitations in the coprecipitation of magnetite at ambient conditions. Whereas previous studies have highlighted the effect of anionic macromolecules and isolated proteins in the biomineralization^37^ and synthetic formation^38^ of magnetite, we underline the significant mechanistic changes induced by a polycation. In contrast to classical wet-chemical routes, where surfactants are used to control nanoparticle morphology by inhibiting the growth of certain crystal faces,^34,35^ the crystallization from amorphous precursors that are shaped by interfacial interaction energy with its subphase magnetite introduces a new approach to control size and morphology of nanoparticles.

## Supporting Information Available

Materials and methods, details on fitting routine and fitted data, and supplementary tables and figures (PDF)
Video SI1 (MPG)

## Supplementary Material

ja1c02687_si_001.pdf

ja1c02687_si_002.mpg
