# Supplementary material for: Wettability of Magnetite Nanoparticles Guides Growth from Stabilized Amorphous Ferrihydrite
Source: J Am Chem Soc. 2021 Jul 15;143(29):10963–9. doi: 10.1021/jacs.1c02687 (PMC8323100; doi:10.1021/jacs.1c02687)
Supplement: Supplementary file 1 — ja1c02687_si_001.pdf [file ja1c02687_si_001.pdf]

Wettability of Magnetite Nanoparticles Guides Growth from Stabilized Amorphous Ferrihydrite

Lucas Kuhrts*[1], Sylvain Prévost[2], Daniel M. Chevrier[1,4], Péter Pekker[3], Oliver Spaeker[1], Mathias Egglseder[1], Jens Baumgartner[1], Mihály Pósfai[3], and Damien Faivre[1,4]

[1]Max Planck Institute of Colloids and Interfaces, Department of Biomaterials, Am Mühlenberg 1, 14476 Potsdam, Germany
[2]Institut Laue-Langevin, 71 avenue des Martyrs, CS 20156, 38042 Grenoble Cedex 9, France
[4]Research Institute of Biomolecular and Chemical Engineering, University of Pannonia, Egyetem u. 10, H8200 Veszprém, Hungary
[4]Aix-Marseille Université, CEA, CNRS, BIAM, 13108 Saint-Paul-lez-Durance, France

May 26, 2021

SI1 Experimental Section

SI1.1 Magnetite precipitation

Magnetite co-precipitation was performed according to the protocol of Kuhrts et al.:[1]
"In brief, magnetite was precipitated in a computer-controlled reaction environment keeping a constant pH via a titration device (Metrohm AG, Titrando 888, 5 mL volumetric cylinder) coupled to a pH electrode (Metrohm, Biotrode). Iron solution was added using a dosing unit equipped with a 1 mL cylinder (Metrohm Dosimat 805). Reactions were performed under nitrogen atmosphere in a 50 mL reaction vessel at a constant temperature of 25.0(1) °C set by a thermostat M3 (Lauda). Magnetite was precipitated from a 0.1 M solution of ferrous ($FeCl_2 \cdot 4\,H_2O$, VWR) and ferric iron ($FeCl_3 \cdot 6\,H_2O$, Sigma-Aldrich) at a ratio $Fe^{2+}/Fe^{3+}=\frac{1}{2}$ that was added at a rate of 3 µL min^{-1} to 30 mL of an alkaline aqueous solution of 0.1 mg mL^{-1} poly(L-arginine hydrochloride) (15 to 70 kDa, Sigma-Aldrich). Depending on the synthesis conditions pH values were set to pH 9, 10 and 11 and controlled by addition of 0.1 M of NaOH solution (1 M, Merck). Homogenization of the precipitation medium was achieved by continuous stirring with a non-magnetic overhead mechanical stirrer. All particles were synthesized for 2 h and afterwards stored at 4 °C under argon atmosphere."

SI1.2 Transmission Electron Microscopy

After 2 h of synthesis an aliquot of 1 mL was centrifuged for 10 min at 10 krpm and re-dispersed in Milli-Q water of appropriate pH. 5 µL of the re-dispersed particles were adsorbed from aqueous suspension for 15 min onto 300 mesh copper grids covered with a Lacey carbon supported graphene layer. Residual solution was removed using a Kimwipe and the grid dried for at least 30 min prior to imaging. Bright field transmission electron microscopy (TEM) and high resolution (HR)TEM were performed on a spherical aberration corrected JEOL ARM 2100 at an acceleration voltage

of 200 kV and an emission current of 15 µA. Evaluation of size distributions for freshly prepared and 7 d aged samples was performed by determination of the projected area of at least 200 particles using ImageJ.

SI1.3 HAADF STEM Tomography

Samples for HAADF STEM Tomography were prepared according to the procedure used in TEM. STEM tomography images were recorded on a FEI Talos equipped with a LaB_6 electron gun. Electrons were accelerated in a 200 kV potential field using a current of 10 µA.
Tomography tilt series on mesocrystals were recorded between 65° to −62° in increments of 1°. Focus and drift corrections were applied manually. Inspect 3D software (Thermo Fisher Scientific) was used for 3D reconstruction of STEM tomography data with SIRT iterative reconstruction method. The visualization of the reconstructed volume was performed in Avizo software (Thermo Fisher Scientific).

SI1.4 *In situ* SAXS

In situ SAXS was measured at the high brilliance beamline ID02 [2] at ESRF – The European Synchrotron (Grenoble, France). For SAXS experiments the X–ray energy was set to 12.5 keV where the highest photon flux is obtained with a U21 undulator. The detector chosen was the CCD Rayonix MX-170HS. Three different sample-to-detector distances were used (1 m, 6.4 m and 30.7 m for synthesis conducted at pH 11. With this setup a q–range of 2×10^{-3} to $5\,nm^{-1}$ was covered and reproducibility ensured through merging the data in absolute scale (see SI). As the 6.4 m sample-to-detector distance setup was sufficient to cover the relevant and accessible q-range to follow particle nucleation, data for synthesis conducted at pH 10 and pH 9 were collected at this configuration. For the *in situ* measurements the synthesis setup described above was installed at the beamline. A 1 mm inner diameter glass tube was used as an intake pipe that was connected using PTFE tubing (inner diameter 1 mm) via a quartz capillary of exactly 2.0 mm inner diameter (Hilgenberg open quartz capillaries, 50 µm wall thickness) using a step motor peristaltic pump (WMC). The dead volume between the beginning of the glass tubing and the end of the quartz capillary was calculated to be 70 µL. To ensure that the old sample was entirely replaced by the new one, an aliquot of 80 µL was sucked from the reaction just before acquisition at different time intervals (20 times every 30 s, 20 times every 60 s, 20 times every 300 s, totalling 2 h and 10 min). To avoid radiation damage, that would alter the sample in the illuminated volume and would cause a progressive degradation of the background (deposit on the capillary walls), the acquisition time was constant and short even for weakly scattering samples, resulting in sub-par statistics at initial stages. Absolute intensity was calculated from water scattering (after subtraction of the empty capillary contribution). The measured water scattering intensity was compared to the theoretically expected scattering level, obtained from water's isothermal compressibility and electronic density ($d\Sigma_{H_2O}/d\Omega = 1.68 \times 10^{-2}\,cm^{-1}$), resulting in a correction factor applied to all SAXS curves. The first file of each dataset was used as background and subtracted from all consecutive frames. Four days of beamtime were awarded with the experiment ID MA3787. Time-dependent SAXS data was fitted using the Matlab-based program SASET [3] adopting analytical expressions found in the SASfit documentation. [4] A sketch of the scattering setup is added at the end of the supporting information in Figure SI12.

SI1.5 Cryo-XAS

Fe K-edge XANES and EXAFS spectra were recorded on the I20-scanning beamline at Diamond (Oxford, UK). We used Si (111) four-bounce monochromator and double-focusing mirrors, giving a beam spot size of 400 µm × 300 µm at the sample position. Data was recorded in fluorescence detection mode, using a 64 element monolithic Ge detector with Xspress4 digital pulse processor. Reference samples were measured in transmission mode using a gas ion chamber for detection. During all measurements, samples were cooled to 77 K using a liquid N_2 cryostat. Spectra were collected

scanning the undulator gap using an energy resolution of 3 eV between 7.0 to 7.1 keV, 1 eV between 7.1 to 7.3 keV and 3 eV between 7.3 to 7.8 keV. Data were averaged, normalized and deglitched using Athena 0.9.26.
The synthesis protocol presented above was adapted to the requirements of the I20-scanning beamline acknowledging the detection limit for iron at incident x-ray energy around 7.1 keV of 30 ppm. Therefore the initial precipitation volume reported above was reduced to 10 % to obtain sufficient signal during the first minutes. Invariance of the resulting particle morphology and size as it is reported in the manuscript against a change in precipitation volume was ensured prior using TEM.
For sample preparation, we synthesized magnetite with and without the addition of poly(L-arginine) and took samples at 5 and 20 min into the synthesis and froze them in Kapton tubes(4.2 mm outer diameter, 0.07 mm wall thickness, 0.8 cm length) using liquid nitrogen. To ensure necessary homogeneous freezing, the Kapton tube was filled with powdered cellulose and 20 µL of the sample was gently pressed through the tube prior to the freezing. Samples were transported to the beamline in liquid nitrogen and transferred to the cryostat using a 3D printed polylactic acid (PLA) sample holder.

SI2 Data Treatment

SI2.1 Treatment of SAXS data

Time-dependent SAXS data was fitted using the Matlab-based program SASET. [3] Time series were fitted consecutively where parameters of t_n were passed on to t_{n+1} using a unified scattering model. All scattering curves at every time point can be analytically described by:

$$\frac{d\Sigma}{d\Omega}(q,t_n) = P_{\mathrm{GenGauss}}(q) + {}^1n_{\mathrm{Sphere}} \cdot P_{\mathrm{Sphere}}(q) \cdot [(1-\xi_{\mathrm{mag}}) + \xi_{\mathrm{mag}} \cdot S_{\mathrm{SW}}(q)] \tag{SI2.1}$$

$P_{GenGauss}(q)$ is the generalized Gaussian coil scattering model, classically used to describe polymer scattering but mathematically congruent to the scattering model of a fractal with the condition of a fractal dimension below 2.6 and a q-resolution lower than a fractal sub-unit which is true for the present system. As input parameters the model takes a gyration radius R_g, the Flory exponent ν — which in these conditions is the reciprocal of the Hausdorff (fractal) dimension $D_f = 1/\nu$, and the forward scattering of the precursor structures $I_{\mathrm{GenGauss}}(q=0)$ in cm^{-1} with the following relation:

$$I_{\mathrm{GenGauss}}(0) = \frac{\rho \cdot M_w \cdot \Delta\mathrm{SLD}_{\mathrm{fh}}^2}{N_A \cdot d_{\mathrm{fh}}^2}$$

where M_w is the molecular mass of ferrihydrite in $\mathrm{g\,mol^{-1}}$ ($[\mathrm{Fe}^{3+}_{8.2}\mathrm{O}^{2-}_{8.5}(\mathrm{OH})^{-}_{7.4} \cdot 3\,(\mathrm{H_2O})]_{\mathrm{n}}^{0.2+}$ [5], $M_w = 773.8 \times n\,\mathrm{g\,mol^{-1}}$), ρ the mass concentration in $\mathrm{g\,cm^{-3}}$, d_{fh} the apparent density ($d_{\mathrm{fh}} = 4.15\,\mathrm{g\,cm^{-3}}$) and $\Delta\mathrm{SLD}_{\mathrm{fh}}$ the contrast: $\mathrm{SLD}_{\mathrm{fh}} = 34.6 \times 10^{10}\,\mathrm{cm^{-2}}$ (and with $\mathrm{SLD}_{\mathrm{water}} = 9.4 \times 10^{10}\,\mathrm{cm^{-2}}$ the resulting contrast is $\Delta\mathrm{SLD}_{\mathrm{fh}} = 25.2 \times 10^{10}\,\mathrm{cm^{-2}}$). Dense magnetite nanoparticles are approximated by a spherical form factor: $P_{\mathrm{Sphere}}(q)$, with an analytically implemented Schulz-Zimm size distribution. As input parameters the model takes the volume fraction, size and size distribution of the magnetite nanoparticles. The number density of these spherical particles is ${}^1n_{\mathrm{Sphere}}$. $P_{\mathrm{Sphere}}(0) = \langle V_{\mathrm{Sphere}}^2 \rangle \Delta\mathrm{SLD}_{\mathrm{mag}}^2$, where $\Delta\mathrm{SLD}_{\mathrm{mag}} = 42.0 \times 10^{10} - 9.4 \times 10^{10} = 32.6 \times 10^{10}\,\mathrm{cm^{-2}}$ with $d_{\mathrm{mag}} = 5.175\,\mathrm{g\,cm^{-3}}$. Using the volume fraction of nanoparticles, as $\Phi = {}^1n_{\mathrm{Sphere}} \cdot \langle V_{\mathrm{Sphere}} \rangle$ and with the monodisperse approximation $\langle V_{\mathrm{Sphere}} \rangle^2 \approx \langle V_{\mathrm{Sphere}}^2 \rangle$, the forward scattering contribution of nanoparticles is

$$I_{\mathrm{Sphere}}(0) = \Phi \cdot \langle V_{\mathrm{Sphere}} \rangle \cdot \Delta\mathrm{SLD}_{\mathrm{mag}}^2 \cdot [(1-\xi_{\mathrm{mag}}) + \xi_{\mathrm{mag}} \cdot S_{\mathrm{SW}}(0)]$$

The total magnetite concentration calculated from the continuous addition of 0.1 M iron solution at $1\,\mu\mathrm{L\,min^{-1}}$ is shown in Figure SI1 considering the reduction of synthesis volume by sampling and evaporation.

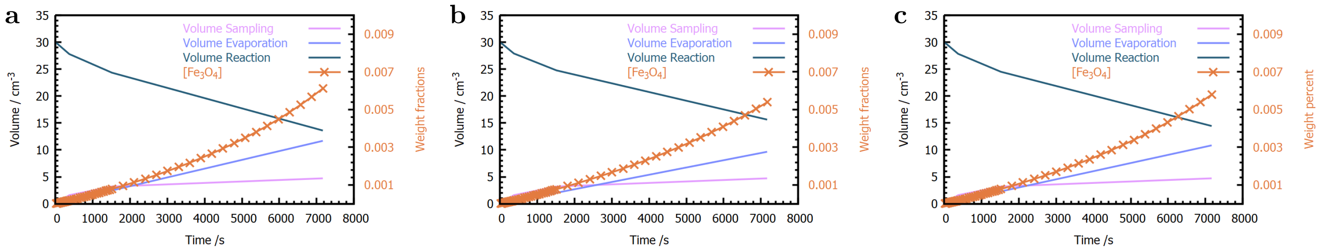

Figure SI1: Solvent reduction through sampling and evaporation during the synthesis and the resulting residual volume in the reactor from which total magnetite concentration is calculated for synthesis at pH 9, pH 10 and pH 11, **a**–**c**, respectively.

Magnetic inter-particle interaction is approximated using the thin square-well pair potential structure factor ($S_{\mathrm{SW}}(q)$) operating on the fraction ξ_{mag} of nanoparticles that has grown above the ferrimagnetic size threshold (attractive interactions originate from magnetic dipole-dipole forces and will be larger for larger particles, but given the narrow size distribution a possible fractionation of nanoparticles between bulk and aggregated particles is not taken into account). As input parameters the model takes a (local effective) packing volume fraction, the depth (in k_BT) and width[1] of the potential well (the particle size is taken as the one from $P_{\mathrm{Sphere}}(q)$). Analytical expressions are found in the SASfit documentation.[4] $S_{\mathrm{SW}}(0)$ is the mean number of particles per chain cluster.

SI2.2 Linear combination fits EXAFS

To quantify the composition of the samples taken after 5 min and 20 min of the synthesis with and without poly(L-arginine), we used linear combination fitting using references of ferrihydrite, magnetite and Fe^{2+} in 0.1 mg mL^{-1} poly(L-arginine) solution. Magnetite was prepared in a 6 h co-precipitation following the protocol described above, while ferrihydrite was prepared following a protocol by Schwertmann.[6] Accordingly, 330 mM KOH were added to 500 mL 0.1 M $Fe(NO_3)_3$ solution until a pH of 7 to 8 was reached. The product was dialyzed against Milli-Q water for 5 d with regular water exchange and consequently lyophilized. Synchrotron powder X-ray diffraction data of the obtained ferrihydrite and magnetite are shown in Figure SI2.

The fitted $k^2\chi(k)$ are shown in Figure SI3 and the corresponding contributions of the reference samples summarized in Table SI1. The data shows that at 5 min ferrihydrite is prevalent in both samples with a contribution of 17 % and 21 % without and with the addition of poly(L-arginine), respectively. After 20 min most of the ferrihydrite (31 %) has transformed to magnetite (68 %) when no poly(L-arginine) is added. A kinetic ferrihydrite stabilization is visible for the sample taken after 20 min with the addition of poly(L-arginine) were 80 % of ferrihydrite and only 17 % of magnetite were calculated to be present.

The R-factor (see Table SI1) given by Athena as a quantification of the goodness of the fits (χ^2 has a similar meaning) is calculated as:

$$R = \frac{\sum(\mathrm{Data} - \mathrm{Fit})^2}{\sum(\mathrm{Data} - \mathrm{Fit})} \qquad \text{(SI2.2)}$$

We find for the early samples with and without poly(L-arginine) a rather large R and visible structuring in the residues of the fits. This may indicate the presence of a condensed iron containing structure which is not reproduced by the reference samples used. We suggest a smaller, transient building unit to be causing this structuring in the early EXAFS samples, which is overshadowed by the signal of magnetite and ferrihydrite at later stages. These structures may be Fe-Keggin clusters that have been suggested to be involved in ferrihydrite formation.[7]

[1] In the case of the thin well potential model, the width is a few percent of the particle's diameter.

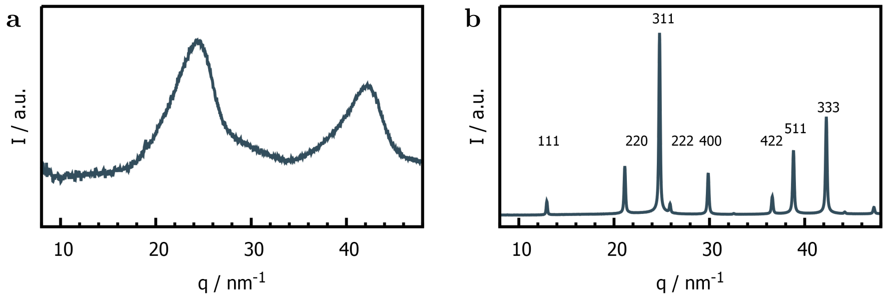

Figure SI2: XRD measurements of reference samples for 2-line ferrihydrite **a** and magnetite **b** used in XAS measurements.

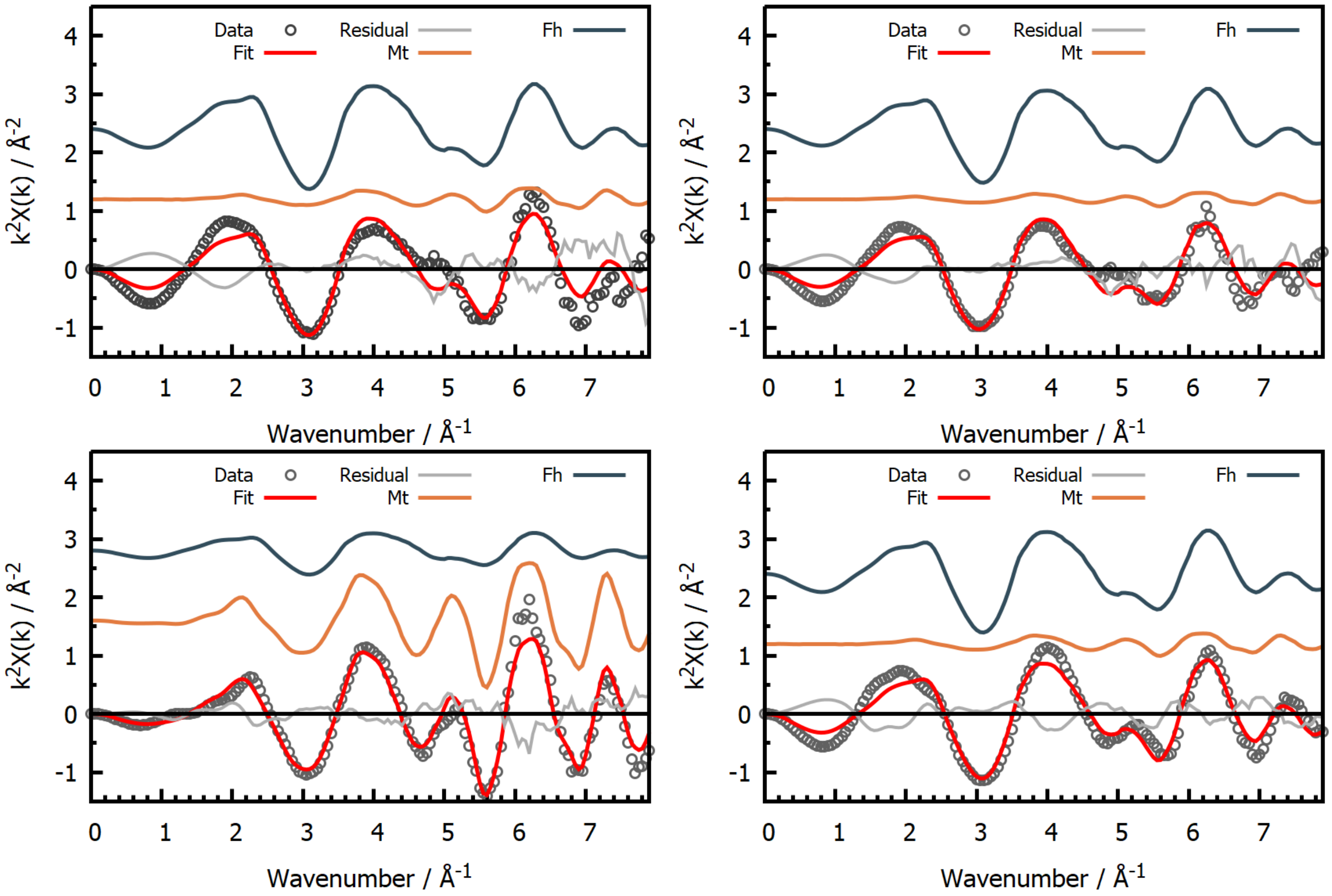

Figure SI3: Linear combination fitting of the EXAFS data with reference samples of magnetite (Mt) and Ferrihydrite (Fh). Data was recorded for samples taken after 5 min (top) and 20 min (bottom) for synthesis in absence (left) and with the addition (right) of poly(L-arginine).

SI2.3 Model Free SAXS Data Analysis

We can confirm a monomer addition mechanism following Huber et al.[8] independent of the scattering model based analysis. Model independent scattering parameters were calculated using SASfit's "Integral Parameters" function, with the Guinier ($I_{\text{Guinier}}(q) = I_{\text{Guinier}}(0) \exp\left[-{(qR_g)^2}/{3}\right]$) and Porod ($I_{\text{Porod}}(q) = a_{\text{Porod}} \cdot q^{-4}$) approximations. We determined parameters in the Guinier region 0.15 to 0.28 nm^{-1} (avoiding the low q regime where a structure factor appears eventually) and in the Porod region 0.83 to 1.11 nm^{-1}. For analysis of the formation mechanism we determined the forward scattering $\frac{d\Sigma}{d\Omega}(q = 0)$ of the nanoparticles, the scattering invariant $\tilde{Q}$, the gyration radius R_g.

$$\tilde{Q} = \int_0^{\infty} I(q)q^2\,dq = \int_0^{q_{min}} I_{\text{Guinier}} q^2\,dq + \int_{q_{min}}^{q_{max}} I_{\text{exp}} q^2\,dq + \int_{q_{max}}^{\infty} I_{\text{Porod}} q^2\,dq = 2\pi^2\Phi(1-\Phi)\Delta\text{SLD}^2 \quad \text{(SI2.3)}$$

$$I(q=0) = \Phi\Delta\text{SLD}^2 V_P \quad \text{(SI2.4)}$$

Table SI1: Results for linear combination fitting of the $k^2\chi(k)$ with reference samples of pure magnetite, ferrihydrite and 0.1 M $FeCl_2$ with poly(L-arginine). A low R-factor is indicative of the goodness of the fit.

Sample	Magnetite / %	Ferrihydrite / %	Fe^{2+}/polyR	R-factor(fit)
5 min **w/ poly(L-arginine)**	17	70	12	0.252
5 min **w/o poly(L-arginine)**	21	78	0	0.271
20 min **w/ poly(L-arginine)**	17	80	2	0.095
20 min **w/o poly(L-arginine)**	68	31	0	0.098

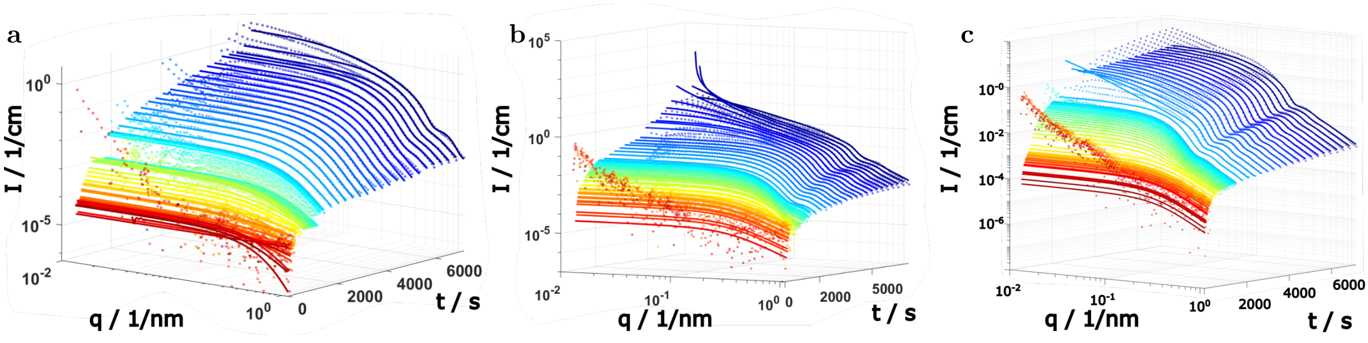

Figure SI4: Plots of full dataset with corresponding best fits for particles prepared at pH 9 (**a**), pH 10 (**b**) and pH 11 (**c**). Datasets for different pH were fitted according to the model presented in Section SI2.1. The data for all pH as well as the corresponding fits are shown in Figure SI4. The thin square well structure factor used to model the particle interaction can well describe the excluded volume effects visible in the mid-q region and can thus optimize the output parameter quality for nanoparticle size and concentration. However, as this structure factor is suited to describe intermediate distance particle-particle interaction the fit quality for the low-q behavior, which corresponds to larger distances, is hampered.

The $I(q = 0)$ corresponds to the weight averaged mass M_W of the ensemble of particles including solid nanoparticles and precursors. Since we are interested in the formation mechanism of only the solid nanoparticles, we determine the $I(q = 0)$ of the particle ensemble only after an hour of synthesis. Here, the forward scattering of the precursors amounts to $1 \cdot 10^{-4} mm^{-1}$. Extrapolating the forward scattering of the particle ensemble to q=0 after 1 h we find a forward scattering of approximately $1\,\mathrm{mm}^{-1}$. The contribution of the precursor particles to the overall forward scattering is 0.01 % and can thus be neglected. To determine the formation mechanism of the solid nanoparticles we can now imagine two mechanisms to take place.

The first mechanism is a particle growth by agglomeration of solid nanoparticles. Let us assume all particles will in a first step agglomerate with one of their neighbors. This will induce a bisection in particle number density, while the volume created by the aggregation of two same sized particles will be twice the initial volume $V_f = 2V_i$. The volume fraction, Φ, of solid nanoparticles, however, will remain constant. We thus write the forward scattering as a function of a constant volume fraction:

$$I(q = 0) = \Phi \Delta \mathrm{SLD}^2 V \tag{SI2.5}$$

substituting the radius of gyration for V with

$$V = \frac{4\pi}{3}\left(\sqrt{\frac{5}{3}} R_G\right)^3 \tag{SI2.6}$$

results in a power law between the forward scattering and the radius of gyration of $I(q = 0)^{1/3} \propto R_G$.

A formation mechanism where a constant fraction of nanoparticles grows by the addition of small invisible "monomer" units, results in an increase in volume fraction, while the number density of the observed particles remains constant. We thus write the forward scattering as a function of the number density, N, of solid particles as:

$$I(q = 0) = N \Delta \mathrm{SLD}^2 V^2 \tag{SI2.7}$$

substituting the radius of gyration for the volume we establish a power-law of $I(q=0)^{1/6} \propto R_G$ for a monomer addition mechanism. Figure SI5 a) shows the radius of gyration as a function of the forward scattering of nanoparticles prepared at ph 9, 10 and 11 respectively. All pH follow a power-law of 1/6 supporting the model based analysis that also predicts a nanoparticle growth mechanism based on monomer attachment. With the extended q-range accessible in our SAXS experiment it is possible to calculate the integrated scattering intensity — via the scattering invariant — which allows for the normalization of the weight averaged mass, giving access to the weight-averaged mass of the solid nanoparticles, M_P:

$$M_P = \frac{2\pi^2}{\tilde{Q}} \frac{d\Sigma}{d\Omega}(q=0) \tag{SI2.8}$$

The particle size expressed as the radius of gyration reveals a power law of $R_G \propto M_P^{\alpha}$ with $\alpha = 1/3$ in the case of solid spherical particles. Figure SI5 b) shows the weight-averaged mass of solid nanoparticles plotted as a function of their forward scattering revealing the expected slope of 1/3, emphasizing the consistency of the presented analysis. The fact that the curves overlay well for all pH further demonstrates that all observed particles have an equal polydispersity and density.

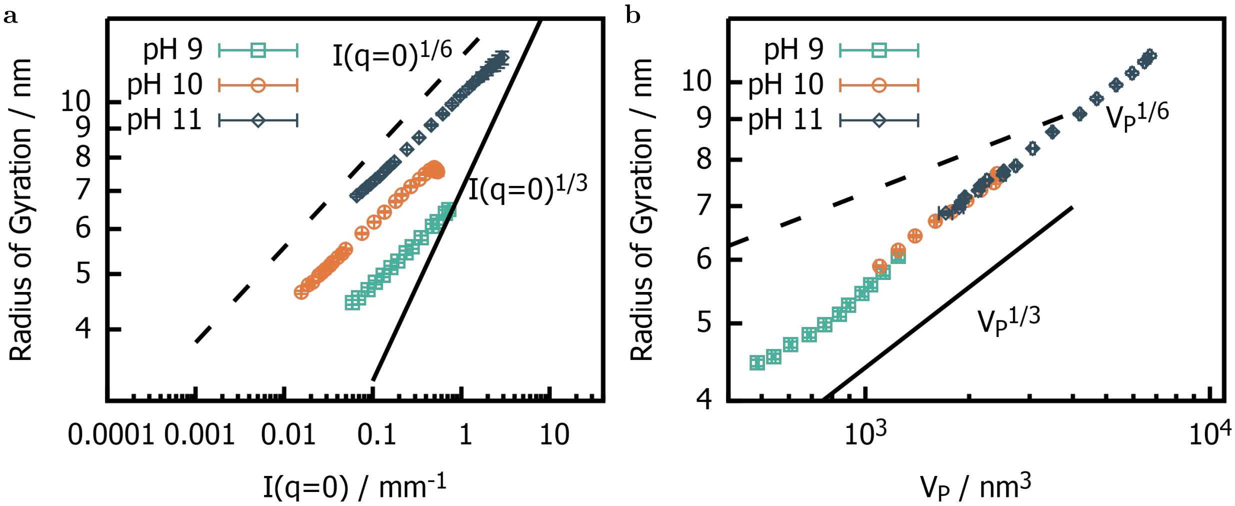

Figure SI5: a–b Scattering parameters from SAXS data determined without the assumption of a particle shape-based scattering model for all pH values. **a** shows the radius of gyration obtained after 1 h of synthesis as a function of the forward scattering of the magnetite particles, which follows a power law of $^1/_6$, indicative of a mechanism of discrete addition of a precursor with negligible scattering intensity. **b** shows the radius of gyration as a function of particle volumes, which follows a power law of $^1/_3$. Indicative of the growth of solid, spherical nanoparticles. The well overlapping curves further indicated an equal particle size distribution of particles with an equal density.

SI2.4 Prediction of the pH dependent contact angle between magnetite and ferrihydrite precursor

The interaction between the precursor and the magnetite surface in water can be relatively quantified using the Young-Laplace equation:

$$\gamma_{\mathrm{Mt,H_2O}} = \gamma_{\mathrm{Pre,Mt}} + \gamma_{\mathrm{Pre,H_2O}} \cos(\theta)$$

An absolute evaluation of the surface energies at the nanoscale as well as a determination of the contact angle is difficult, if not impossible. Nevertheless, deducing a relative effect of a change in pH on a change in contact angle is possible.

$$\Delta\gamma_{\mathrm{Mt,H_2O}}(\mathrm{pH}) = \Delta\gamma_{\mathrm{Pre,Mt}}(\mathrm{pH}) + \Delta\gamma_{\mathrm{Pre,H_2O}}(\mathrm{pH}) \cos(\Delta\theta(\mathrm{pH}))$$

We determine that the most dominant influence of the pH on the surface energies of magnetite and ferrihydrite is induced by the deprotonation of the surface hydroxyl groups, increasing the surface charge density and therefore decreasing the surface tension with water. Due to similar surface chemistry of magnetite and the precursor we can further assume that the change in surface tension between magnetite and water, and ferrihydrite and water will change alike with pH.

$$\Delta\gamma_{\mathrm{Mt,H_2O}}(\mathrm{pH}) = \Delta\gamma_{\mathrm{Pre,H_2O}}(\mathrm{pH})$$

We can thus write the Young-Laplace equation as:

$$\Delta\gamma_{\mathrm{Mt,H_2O}}(\mathrm{pH}) = \Delta\gamma_{\mathrm{Pre,Mt}}(\mathrm{pH}) + \Delta\gamma_{\mathrm{Mt,H_2O}}(\mathrm{pH})\cos(\Delta\theta(\mathrm{pH}))$$

Solving for the contact angle, θ, gives us the relation between the contact angle and the pH dependent surface tensions:

$$\Delta\theta(\mathrm{pH}) \approx \cos^{-1}\left(1 - \frac{\Delta\gamma_{\mathrm{Pre,Mt}}(\mathrm{pH})}{\Delta\gamma_{\mathrm{Mt,H_2O}}(\mathrm{pH})}\right)$$

SI3 Magnetite Co-precipitation in absence of additives

Magnetite is formed via co-precipitation of ferrous and ferric chloride in aqueous alkaline solution. The hydrolysis reaction can be expressed as:

$$2\,\mathrm{FeCl_{3(aq.)}} + \mathrm{FeCl_{2(aq.)}} + 8\,\mathrm{NaOH_{(aq.)}} \longrightarrow \mathrm{Fe_3O_{4(s)}} + 4\,\mathrm{H_2O} + 8\,\mathrm{NaCl_{(aq.)}}$$

A comprehensive study on the formation of magnetite nanoparticles produced under the same conditions as in this work was done by Baumgartner et al.[9]. They have presented an in depth analysis of the structural evolution of magnetite nanoparticles and the underlying thermodynamics of possible nucleation pathways. The kinetics of magnetite nucleation was modeled with a linear ($\alpha = 1$) reaction limited growth $R^{\alpha}(t) - R^{\alpha}_{(t=0)} = kt$ from finite size seeds, $R_{(t=0)}$, where k is the kinetic rate constant and t the time. The growth of the nanoparticles was studied as a function of pH and an inverse proportionality of the rate constant with pH was identified. This rate is proportional to the probability of a seed to grow beyond its critical radius crossing the free energy barrier ($k \propto \exp(-\Delta G_c/k_B T)$). When the pH is increased, the deprotonation induced charging of the magnetite/water interface induces a lowering in surface tension. This lowering in surface tension results in a lower free energy barrier. Thus, smaller seeds will be formed faster when the synthesis pH is increased. Thus, from a unit volume iron solution we will obtain more and smaller seed particles at increased pH, decreasing the radial growth rate of each individual particles, as summarized in Figure SI6.

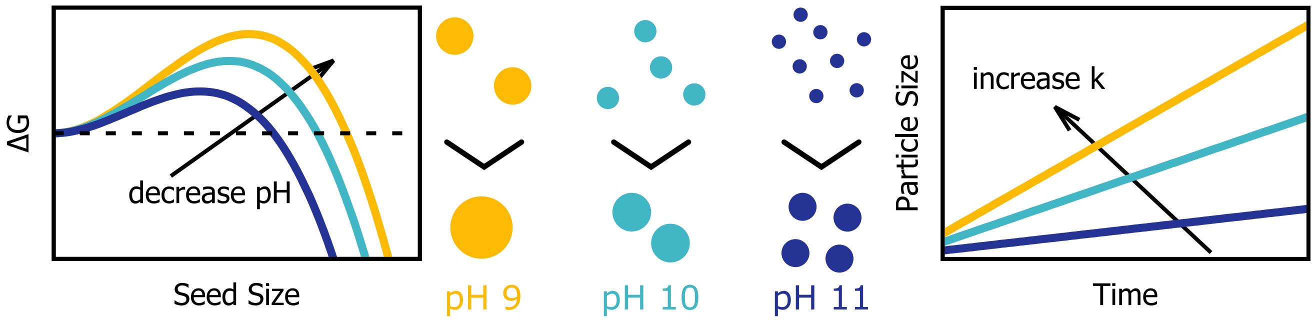

Figure SI6: Free energy as a function of pH. With increasing pH the activation energy and the critical size for stable seeds decreases. We thus form from a unit volume iron solution more but smaller particles at higher pH. Looking at a single particle, which is supposed to grow by the consumption of other particles, a decrease in the reaction rate per time unit is suggested.

SI3.1 Magnetite precipitation in the presence of salts

Magnetite was precipitated from ferrous and ferric chloride according to Section SI1.1. This procedure was altered reducing the reaction volume to 10 mL and reducing the feeding rate to 1 µL min^{-1} as it was reported in Kuhrts et al.[1] Further we added 10 mM of either NaBr, NaCl or KCl to the initial reaction volume before magnetite precipitation was started. The 10 mM correspond to an increase in counter ion concentration after 2 h due to the consistent addition of 0.1 M iron solution at a feeding rate of 1 µL min^{-1} to a reaction volume of 10 mL. Representative TEM micrographs of particles prepared at pH 11 (Figure SI7) only show nanoparticles ill-defined morphology. The electron diffraction patterns (Figure SI7 inset) were indexed according to the inverse spinel structure of magnetite, indicating that the addition of salt does not significantly alter the phase of the nanoparticles.

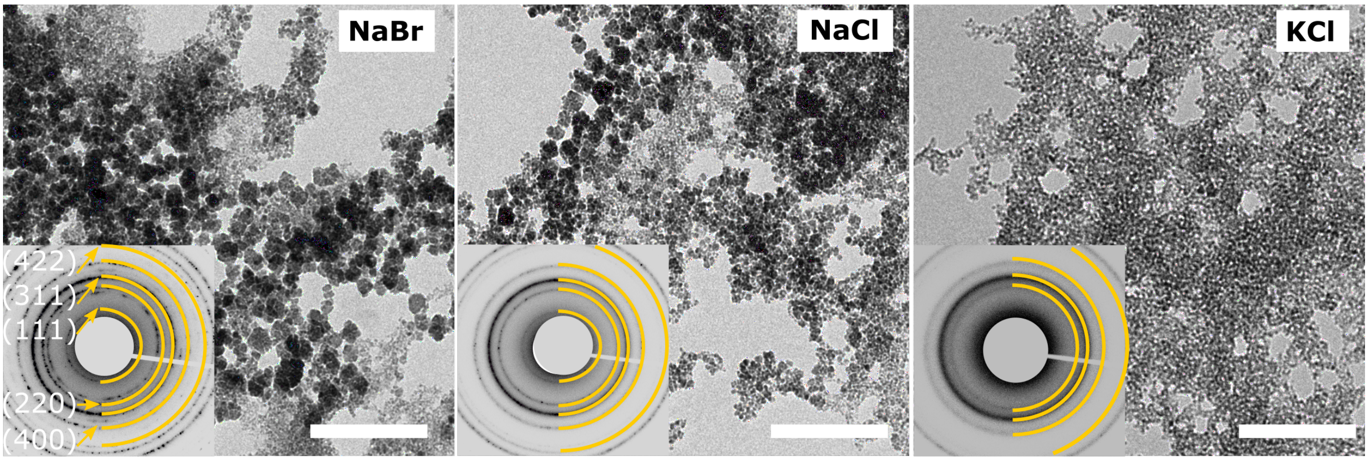

Figure SI7: TEM images of magnetite nanoparticles synthesized at pH 11 with an initial addition of 10 mM NaBr, NaCl or KCl. Electron diffraction patterns (inset) were indexed according to the inverse spinel structure of magnetite. Scale bar represents 200 nm.

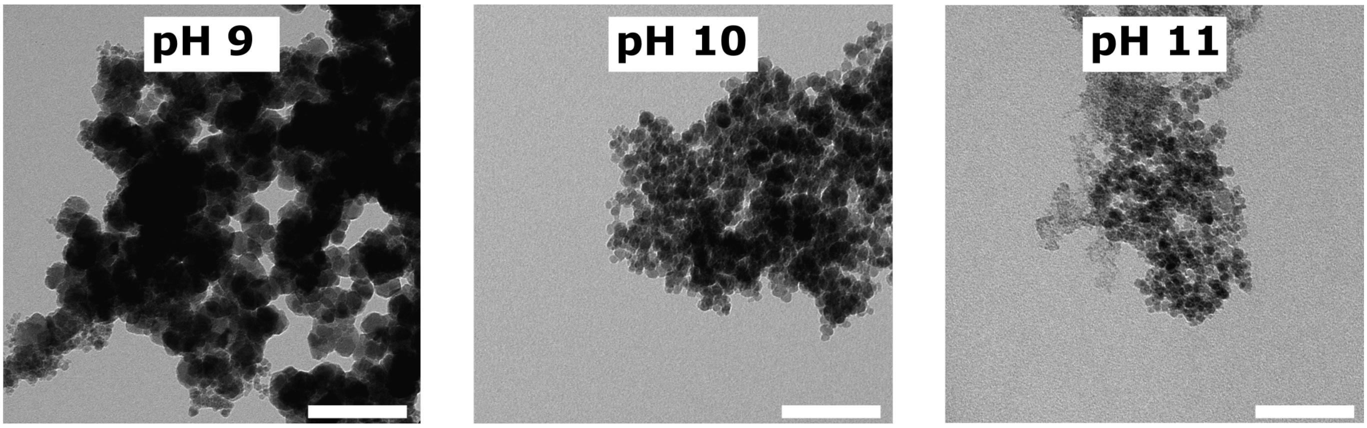

Figure SI8: TEM images of magnetite nanoparticles prepared in absence of poly(L-arginine) at pH 9, 10 and 11. Scale bar represents 100 nm.

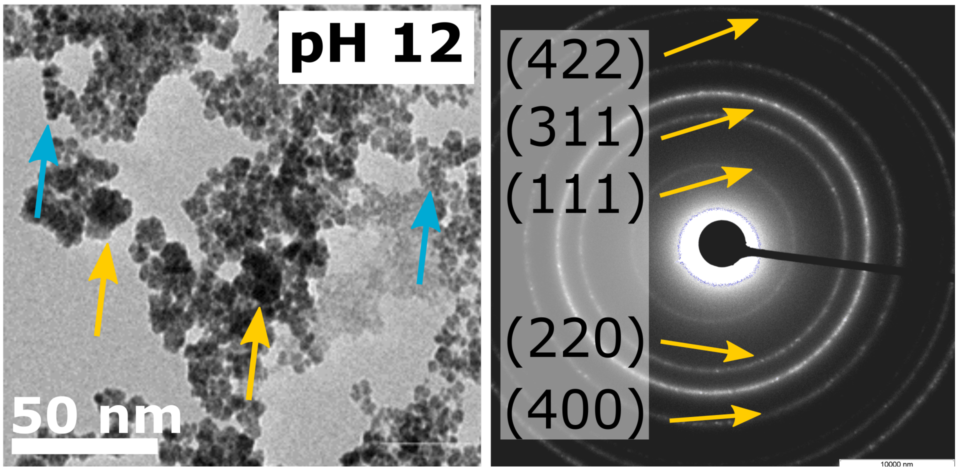

Figure SI9: TEM images (left) of magnetite nanoparticles precipitated at pH 12. Yellow arrows indicate sub-structured nanoparticles that, however, lack a defined shape. Blue arrows indicate small magnetite nanoparticles with dimensions of 1 to 2 nm. This size corresponds to the size of the 4 nm low-density ferrihydrite precursors when self-condensed to form a magnetite nanoparticle. The electron diffraction pattern (right) can be indexed according to the inverse spinel structure of magnetite.

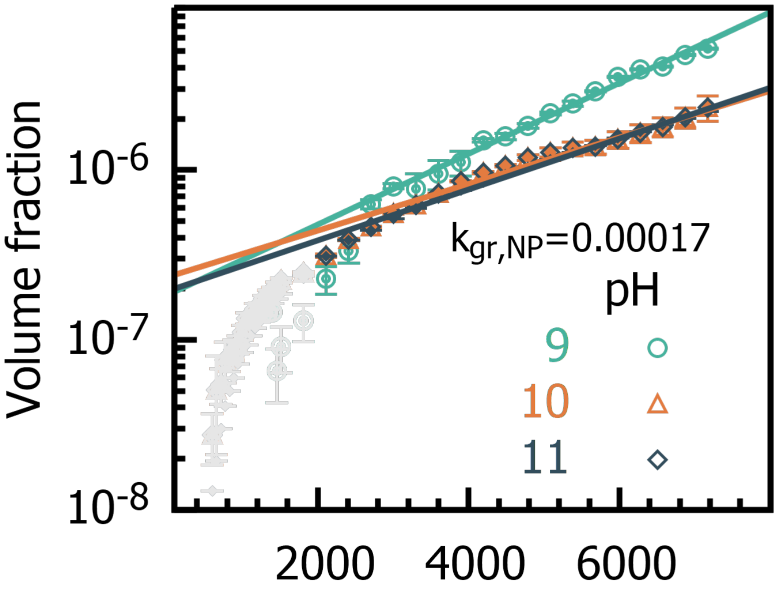

Figure SI10: Volume fractions calculated from fits for solid magnetite nanoparticles prepared at pH 9, 10 and 11, respectively. Here, $k_{gr,NP}$ is the rate constant of the increase of the particle volume fraction. Early time points, where the scattering signal of the solid nanoparticles cannot be easily discriminated from precursor scattering are greyed-out

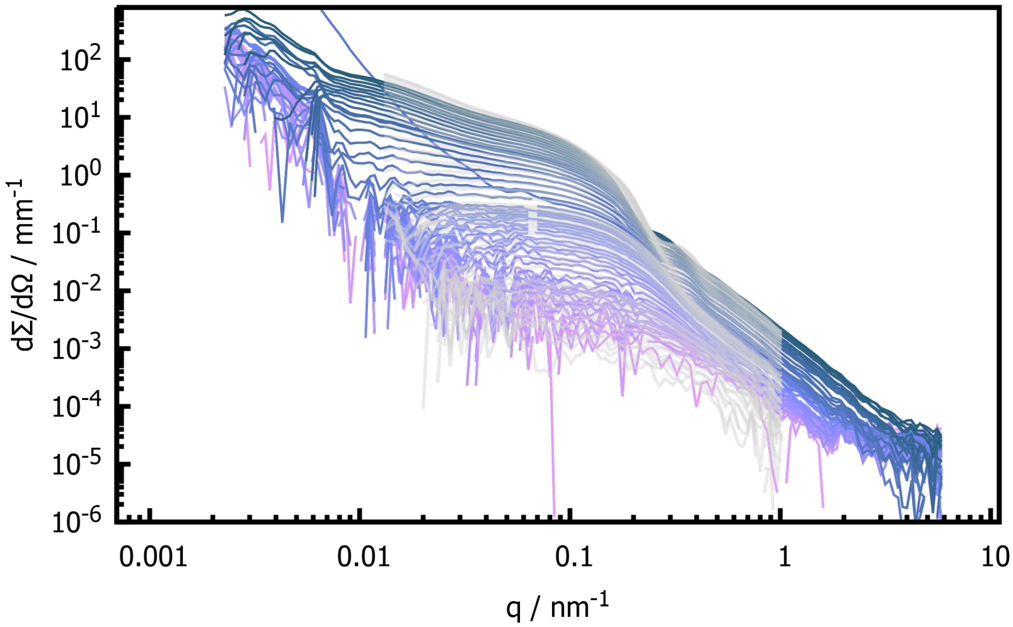

Figure SI11: *in situ* SAXS data of synthesis conducted at pH 11. Data was acquired at 3 different sample-to-detector distances (1 m, 6.4 m and 30.7 m). The colored curves show a merged dataset at 1 m and 30.7 m, while the 6.4 m measurement (grey curve) is overlayed. Good overlap of the three data sets suggest a high reproducibility of the synthesis and the measurements, with only a small change in mean radius.

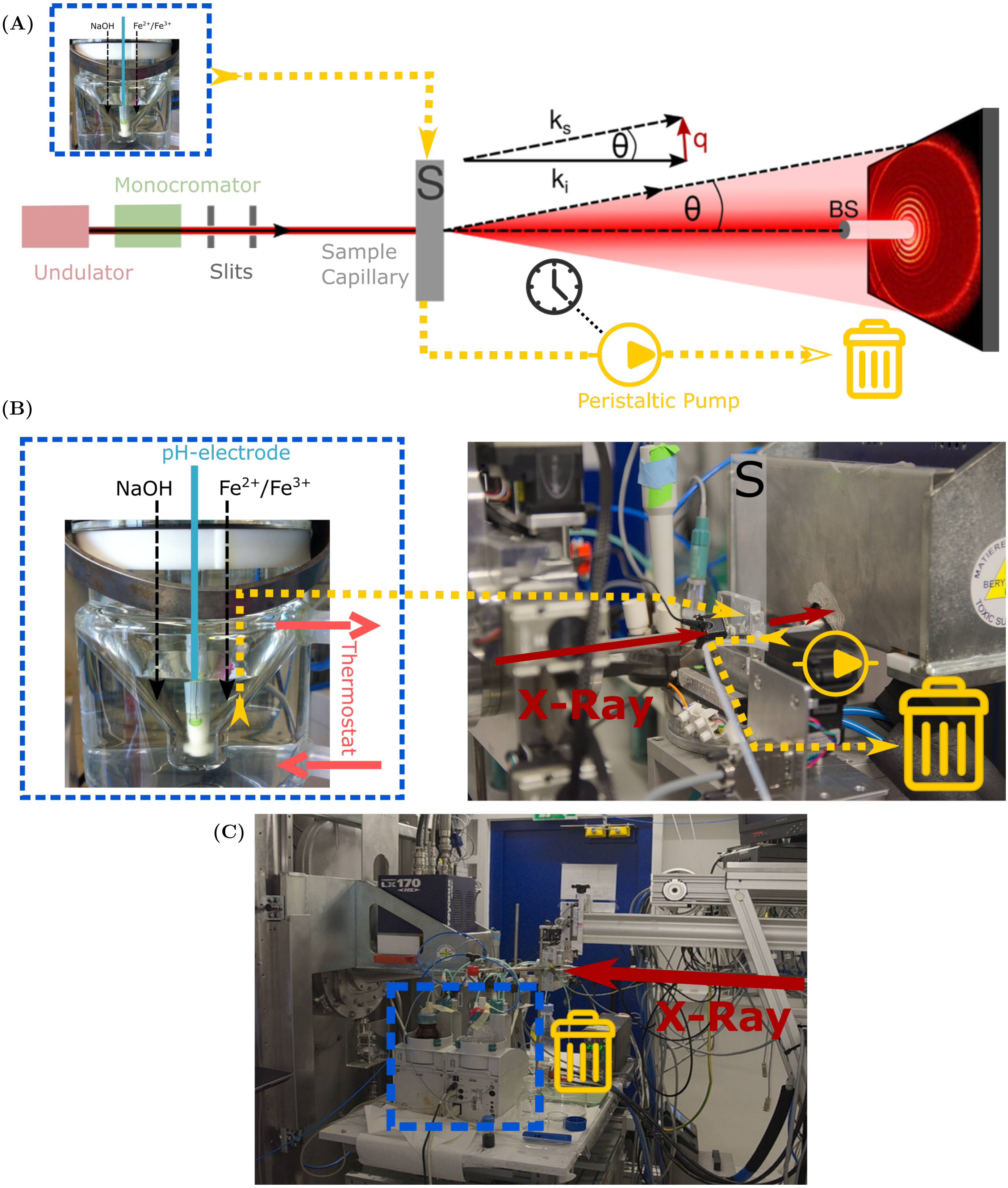

Figure SI12: (A) Schematic of the *in situ* SAXS experimental setup. **(B)**–**(C)** show photographs of the experimental setup with the most important parts highlighted according to their icons in sketch **(A)**

References

[1] Kuhrts, L., Tarakina, N. V., Hirt, A. M., Mac, E. & Faivre, D. Shaping Magnetite with Poly-L-arginine and pH: From Small Single Crystals to Large Mesocrystals. *Journal of Physical Chemistry Letters* **10**, 5514–5518 (2019). URL http://doi.org/10.1021/acs.jpclett.9b01771.

[2] Narayanan, T. *et al.* A multipurpose instrument for time-resolved ultra-small-angle and coherent X-ray scattering. *Journal of Applied Crystallography* **51**, 1511–1524 (2018).

[3] Muthig, M., Prevost, S., Orglmeister, R. & Gradzielski, M. SASET : a program for series analysis of small-angle scattering data. *Journal of Applied Crystallography* **46**, 1187–1195 (2013).

[4] Breßler, I., Kohlbrecher, J. & Thünemann, A. F. *SASfit*: a tool for small-angle scattering data analysis using a library of analytical expressions. *Journal of Applied Crystallography* **48**, 1587–1598 (2015). URL https://doi.org/10.1107/S1600576715016544.

[5] Michel, F. M. *et al.* Ordered ferrimagnetic form of ferrihydrite reveals links among structure, composition, and magnetism. *Proceedings of the National Academy of Sciences* **107**, 2787–2792 (2010). URL https://www.pnas.org/content/107/7/2787. https://www.pnas.org/content/107/7/2787.full.pdf.

[6] Schwertmann, U. & Cornell, R. *The Iron Oxides* (Wiley-VCH, 2004). URL http://doi.org/10.1002/3527602097.

[7] Weatherill, J. S. *et al.* Ferrihydrite formation: The role of Fe13 Keggin clusters. *Environmental Science and Technology* **50**, 9333–9342 (2016).

[8] Liu, J. *et al.* Evaluation of the particle growth of amorphous calcium carbonate in water by means of the Porod invariant from SAXS. *Langmuir* **26**, 17405–17412 (2010).

[9] Baumgartner, J. *et al.* Nucleation and growth of magnetite from solution. *Nature Materials* **12**, 310–314 (2013). URL http://www.nature.com/doifinder/10.1038/nmat3558. NIHMS150003.
